# Dual-resistance respiratory muscle training with a novel individualized respiratory exercise device: a randomized controlled trial

**DOI:** 10.1007/s11845-026-04392-1

**Published:** 2026-04-21

**Authors:** Hikmet Ucgun, Buket Akinci, Cigdem Emirza Cilbir, Ozge Ertan Harputlu, Busra Ulker Eksi, Goksen Kuran Aslan, Esen Kiyan

**Affiliations:** 1 Department of Physiotherapy and Rehabilitation Faculty of Health Sciences, Istanbul Atlas University, Anadolu St. No:40, Istanbul, 34408 Turkey; 2 Department of Physiotherapy and Rehabilitation (English), Faculty of Health Sciences, Biruni University, Istanbul, Turkey; 3 Institute of Public Health and Chronic Diseases, Health Institutes of Turkiye, Ankara, Turkey; 4 Department of Physiotherapy and Rehabilitation (English), Faculty of Health Sciences, Istanbul Kent University, Istanbul, Turkey; 5 Department of Physiotherapy and Rehabilitation Faculty of Health Sciences, Biruni University, Istanbul, Turkey; 6 Department of Physiotherapy and Rehabilitation Faculty of Health Sciences, Istanbul University-Cerrahpasa, Istanbul, Turkey; 7 Department of Chest Diseases Faculty of Medicine, Istanbul University, Istanbul, Turkey

**Keywords:** Dynamic hyperinflation, Expiratory muscle training, Functional capacity, Inspiratory muscle training, Respiratory muscle training, Ventilatory efficiency

## Abstract

**Background:**

Respiratory muscle training (RMT) is known to enhance exercise performance and respiratory efficiency; however, the optimal approach and device design for healthy adults remain underexplored. This study aimed to investigate the effects of a novel individualized respiratory exercise device that integrates inspiratory and expiratory muscle training with real-time visual feedback on pulmonary function, respiratory muscle strength, and functional capacity in healthy adults.

**Methods:**

In this prospective, single-blinded, randomized controlled trial, 40 healthy adults aged 18–65 were randomly assigned to an experimental group (EG; novel individualized respiratory exercise device) or a control group (CG; Threshold® IMT + PEP devices). Both groups trained 5 days per week for 8 weeks at 40% of their baseline maximal inspiratory pressure (MIP) and maximal expiratory pressure (MEP). Outcome measures included spirometry, MIP, MEP, and 6-minute walk test (6MWT) parameters, including inspiratory capacity change (ΔIC), minute ventilation (VEpeak), and dynamic hyperinflation.

**Results:**

Both groups showed significant increases in MIP and MEP (p<0.001), with no intergroup differences. No significant changes were observed in FVC%, FEV1%, or FEF25-75% (p>0.05). However, 6MWT distance improved significantly in both groups (p<0.001), with greater gains in the EG (p=0.024, ηp²=1.03). VEpeak increased significantly only in the EG (p=0.013), accompanied by a significant group-by-time interaction for ΔIC (p<0.001, ηp²=3.40), indicating reduced dynamic hyperinflation.

**Conclusion:**

Both the novel and conventional RMT devices effectively improved respiratory muscle strength and functional capacity in healthy adults. However, the novel individualized respiratory exercise device provided superior improvements in ventilatory efficiency and inspiratory capacity. These findings suggest that combined inspiratory–expiratory training with individualized load adjustment and visual feedback may offer a more efficient and engaging method for optimizing ventilatory mechanics and overall exercise performance in health and rehabilitation settings.

## Introduction

Respiratory muscle fatigue caused by deconditioning has been reported to result in decreased physical performance even in healthy individuals [1]. Numerous studies have demonstrated that respiratory muscle training (RMT) enhances exercise performance in healthy populations [2]. This improvement is attributed to delayed onset of respiratory muscle fatigue, redistribution of blood flow from respiratory to peripheral muscles, and reduced perception of dyspnea or peripheral fatigue during exertion [2]. Evidence indicates that inspiratory muscle training (IMT) primarily improves inspiratory muscle strength and endurance, while expiratory muscle training (EMT) enhances expiratory pressure generation [3]; however studies investigating the optimal training modalities and individualized resistance adjustments in healthy adults remain underexplored.

Commercially available respiratory muscle training devices are generally classified as inspiratory, expiratory, or combined systems and are often complemented with devices that enhance ventilation, such as incentive spirometers [4, 5]. However, most conventional devices are limited to a single function, provide a narrow range of resistance, or lack real-time visual feedback to guide training performance [6]. Moreover, few studies have explored the isolated effects of EMT or its combined use with IMT in healthy adults [7]. These limitations underscore the need for multifunctional, feedback-based respiratory training solutions that can be adapted to individual performance levels, offering both clinical and economic advantages for widespread implementation.

Combined inspiratory and expiratory muscle training may provide a more comprehensive physiological stimulus by engaging both phases of the respiratory cycle, thereby improving ventilatory coordination and efficiency [2, 7]. In contrast to single-mode devices, dual-resistance training may facilitate more balanced respiratory muscle activation [3, 7]. Furthermore, individualized resistance adjustment allows training intensity to be aligned with each participant’s baseline respiratory capacity, which is essential for applying the overload principle and achieving optimal adaptation [8]. Real-time visual feedback may further enhance training effectiveness by improving motor learning, breathing pattern control, and exercise adherence through immediate performance-related feedback [6].

**Fig. 1:**
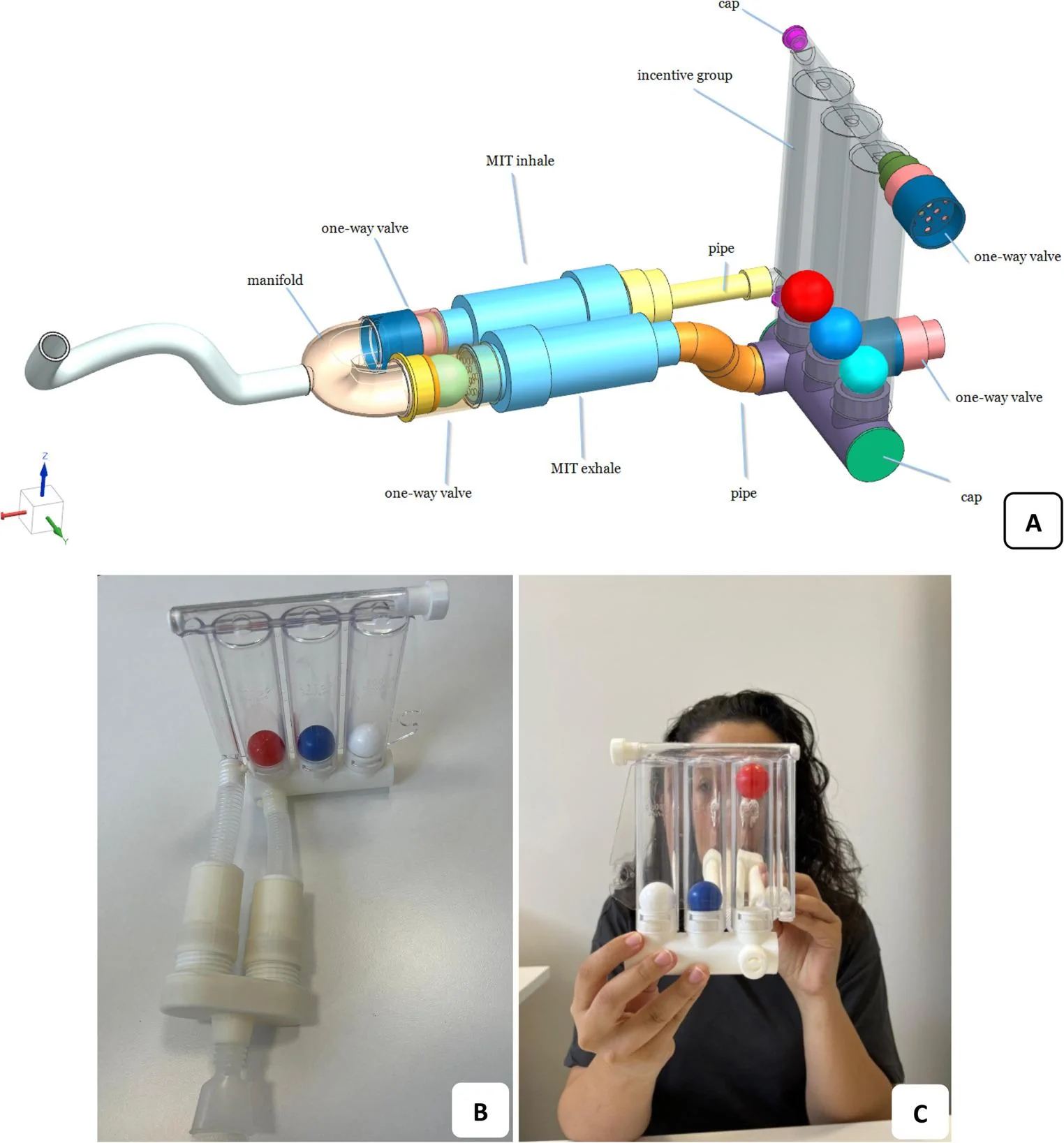
(**A**) The design, (**B**) prototype, and (**C**) practical application of the novel individualized respiratory exercise device

The novel individualized respiratory exercise device integrates the functional principles of incentive spirometry with both IMT and EMT within a single system. It allows users to perform combined respiratory muscle exercises with a broad range of adjustable loads and provides visual feedback on performance in real time (Fig. 1). This personalized approach is expected to enhance user engagement, training adherence, and overall efficacy by aligning resistance levels with each individual’s respiratory capacity. Therefore, the present study aimed to investigate the effects of the novel individualized respiratory exercise device on pulmonary function, respiratory muscle strength, and functional capacity in healthy adults.

## Materials and methods

### Study design and subjects

This prospective, single-blinded study was conducted as a randomized controlled clinical trial between January 2024 and May 2025. Healthy individuals were invited to Department of Physiotherapy and Rehabilitation at Biruni University via an online invitation, and 40 healthy adults aged 18–65 were included in the study. The inclusion criteria were being aged between 18 and 65 years and answering “No” to the health questions in the Physical Activity Readiness Questionare for Everyone [9]. The exclusion criteria were being an amateur or professional athlete who engages in sport-specific training at least twice a week, having a history of spontaneous or trauma-related pneumothorax, pathologies related to the middle ear, or any chronic illness, and receiving routine medical treatment for any reason other than vitamin supplementation.

Eligible participants were randomly assigned to two groups using a computer-based randomisation program by an independent researcher not involved in the assessments or interventions. Allocation concealment was ensured using sealed opaque envelopes. The experimental group (EG, *n* = 20) received the novel individualized respiratory exercise device, while the control group (CG, *n* = 20) received Threshold^®^ IMT (Respironics Inc., USA) and Threshold™ PEP (Respironics Inc., USA). All outcome measurements were carried out by a researcher who was blinded to the group assignments.

The study was approved by the Biruni University Ethics Board (approval number: 2015-KAEK-682201), and the study was registered to the ClinicalTrials.gov website (registration number: NCT06245928). The study was conducted on the ethical principles for human research as outlined by the Declaration of Helsinki and the written informed consent was obtained from all participants.

### Outcome measurements

Spirometry and the 6-minute walk test (6MWT) were administered utilizing the Spiropalm 6MWT^®^ device (COSMED, Italy). Spirometry was conducted using a standard mouthpiece connected to a flow sensor, following the guidelines of the American Thoracic Society (ATS) and the European Respiratory Society (ERS) [10]. Forced vital capacity (FVC), forced expiratory volume in one second (FEV_1_), forced mid-expiratory flow (FEF_25−75_), and peak expiratory flow (PEF) were measured. The percentage of predicted spirometry values were calculated based on the Global Lung Function Initiative 2012 reference standards [11].

Maximum inspiratory pressure (MIP) and maximum expiratory pressure (MEP) were measured using a mouth pressure meter (MicroRPM; MicroMedical Carefusion Micromedical, The United States) [12]. The maximum value from three efforts, each varying by less than 5%, was recorded for both inspiratory and expiratory pressures. Subjects were allowed to rest for approximately one minute between attempts.

The 6MWT was conducted in accordance with ATS guidelines, [13] with the participant wearing a silicone face mask connected to the Spiropalm 6MWT^®^ device (COSMED, Italy). Prior to testing, inspiratory capacity (IC) was measured in accordance with the ATS/ERS guidelines [10]. IC was measured again immediately after the 6MWT. The change in IC from rest to peak exertion (ΔIC) was calculated, with a decrease of 100 ml or more in IC (−ΔIC ≥ 100 ml) being classified as dynamic hyperinflation (DH) [14]. A less negative or more positive ΔIC value was interpreted as a reduced decrease in inspiratory capacity during exercise. The Spiropalm 6MWT^®^ device continuously measured and recorded minute ventilation (VE), respiratory rate (RR), heart rate, and pulse oxygen saturation (SpO_2_) throughout the 6-minute walk test. The change in respiratory rate (ΔRR) during the 6MWT was calculated by subtracting the baseline RR from the RR_peak_. Additionally, the device automatically determined the maximum voluntary ventilation (MVV) as 40 times the participant’s measured FEV_1_. Breathing reserve (BR) was calculated by the device using the formula (MVV−VE_peak_)/MVV, where a BR of less than 30% was considered indicative of a ventilatory limitation to exertion. The 6MWT distance was recorded both as the actual value in meters and as a percentage of the predicted value, based on the prediction equation established for healthy subjects [15]. The Modified Borg Scale was also used to assess dyspnea and fatigue at the beginning and end of the 6MWT.

### Interventions

The development, calibration, and preliminary clinical evaluation of the novel individualized respiratory exercise device have been previously described [16]. In that study, the device underwent biomedical calibration using a differential pressure manometer, demonstrating a wide and reliable pressure range for both inspiratory and expiratory loading. Additionally, pilot testing confirmed its usability and safe application in healthy individuals.

The participants in the EG trained with the novel individualized respiratory exercise device for 5 days in a week for 8 weeks. The training intensity was set to 40% of the participants’ baseline MIP and MEP measurements, which have been shown in the literature to be the lowest training threshold capable of producing a clinical effect in healthy individuals [17]. Participants were asked to rest after 6 breathing cycles and repeat a total of 36 breathing cycles (6 sets) in each session. In each set, the rest period between repetitions was gradually reduced from 60 to 45, 30, 15, 10, and 5 s. Participants were able to perform both inspiratory and expiratory respiratory muscle training in a single breathing cycle. Progression was increased by 10% weekly, with the perceived exertion level being in the range of 4–6 according to the Modified Borg Scale.

The participants in the CG trained with the Threshold^®^ IMT (Respironics; Cedar Grove, NJ, USA) and Threshold™ PEP (Respironics; Cedar Grove, NJ, USA) devices for 5 days in a week for 8 weeks. The training intensity was set to 40% of the participants’ baseline MIP and MEP measurements. Participants were asked to perform a total of 36 repetitions each for inspiratory and expiratory muscle training, consisting of 6 sets of 6 repetitions each. In each set, the rest period between repetitions was gradually reduced from 60 to 45, 30, 15, 10, and 5 s. Progression was increased by 10% weekly, with the perceived exertion level being in the range of 4–6 according to the Modified Borg Scale. If the training threshold exceeds the upper pressure limits of the Threshold^®^ IMT + Threshold™ PEP devices, the training intensity is maintained at the upper limit [18].

Progression was increased by 10% weekly, and all participants were able to tolerate this progression. Therefore, the planned increases were applied consistently across all individuals throughout the intervention period. Training intensity and progression were standardized across both groups using the same relative loading principles. Since all participants tolerated the predefined progression, equivalent increases in training load were achieved in both groups, ensuring a comparable training stimulus. The participants in both groups were instructed to keep a diary for home sessions to improve adherence to exercise program, and it was controlled at the end of the 8 weeks. Adherence (%) of both group was defined as the ratio of the completed sessions to total sessions, which was calculated as “(completed sessions)/(total sessions) multiplied by 100.”

In addition to both training programs, participants in both groups were recommended to engage in physical activity. Participants were instructed to walk for an average of 30 min at least 5 days a week at an intensity level of 4–6 on the Modified Borg Scale [19].

### Statistical analysis and sample size

Statistical analysis was conducted using IBM SPSS v.26 (SPSS Inc.). The normality of the distribution of data was analyzed using the Shapiro–Wilk Test. Categorical variables were compared between groups using the χ^2^ test. Independent Samples T-test or Mann–Whitney U test was used for between-group comparisons depending on the distribution properties of the data. A repeated measure analysis of variance (ANOVA) was used for the analysis of intragroup changes. A mixed ANOVA (repeated-measures analysis of variance with between-subjects factor) was used to analyze whether the effects of interventions differed between the groups. The results were considered significant with p values < 0.05. Complementarily, the effect size was calculated by partial eta-squared (ηp^2^) with small, medium, and large effect sizes classified as 0.01, 0.06, and 0.14, respectively [20].

The G*Power 3.1 (Universitaet Dusseldorf, Germany) software was used for the sample size calculation [21]. Due to the limited availability of prior randomized controlled trials reporting between-group effect sizes for combined inspiratory and expiratory muscle training in healthy individuals, the sample size estimation was based on a previously reported correlation between respiratory muscle strength and functional capacity. This approach was used as a proxy to estimate the expected magnitude of physiological change. Based on the results of a study in the literature, we estimated a sample size of 20 adults for each group [22]. The sample size calculation was performed based on a correlation coefficient of *r* = 0.408 between respiratory muscle strength and 6MWT distance, with 85% power and a two-tailed α level of 0.05. The number of participants was calculated with a 10% increase in sample size in case of any drop-outs. A post-hoc power analysis was conducted based on the primary outcome (6MWT distance) and the observed group-by-time interaction effect, indicating that the study had sufficient statistical power (power > 80%).

## Results

**Fig. 2:**
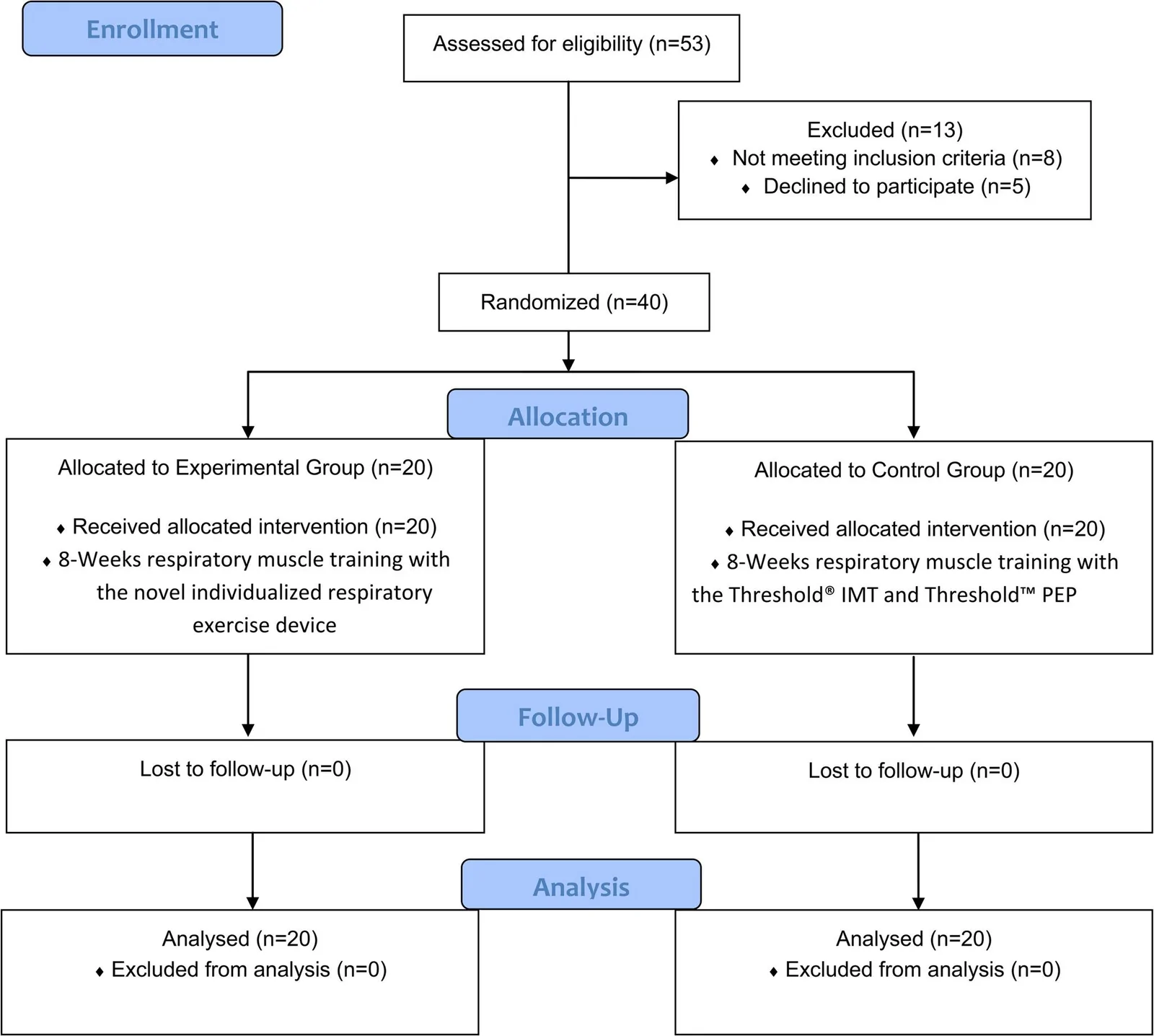
The flowchart of the study

Fifty-three healthy adults were assessed for eligibility. Forty adults who met the inclusion criteria were included in this study and randomized. All 40 participants adhered fully to the training protocol, with no dropouts, and were included in the final data analysis (Fig. 2).

The comparison of the demographic characteristics of the participants is given in Table 1. Demographic variables did not differ significantly between the groups (*p* > 0.05). Adherence to training program were similar and quite high in both groups (*p* = 0.174).

**Table 1:** The demographic characteristics of the participants

	Experimental Group (*n* = 20)	Control Group (*n* = 20)	*p* value
Age (years)	29.20 ± 3.28	30.26 ± 4.35	0.391
Gender			
Female	12 (60%)	13 (65%)	0.584
Male	8 (40%)	7 (35%)	
Body composition			
Body mass index (kg/m^2^)	22.91 ± 2.34	22.48 ± 2.69	0.594
Smoking			
Active smoker	8 (40%)	4 (20%)	0.093
Non-smoker	12 (60%)	16 (80%)	
Employment status			
Working	19 (95%)	18 (90%)	0.877
Not working	1 (5%)	2 (10%)	
Education level			
Illiterate	0	0	1.000
Primary	0	0	
Secondary	0	0	
University	20 (100%)	20 (100%)	
Adherence to training program	88.27 ± 3.62	83.97 ± 5.16	0.174

Data are presented as mean ± standard deviation or n (%)

Table 2 presents a comparison of the baseline values in pulmonary function, respiratory muscle strength and functional capacity between EG and CG. There were no significant differences between the baseline values of the groups (*p* > 0.05).

**Table 2:** Comparison of baseline values in pulmonary function, respiratory muscle strength and functional capacity between the experimental and the control groups

	Experimental Group (*n* = 20)	Control Group (*n* = 20)	*p* value	95% Cl
Pulmonary function				
FVC (%pred)	91.47 ± 9.78	96.00 ± 8.47	0.381	-3.17–8.11
FEV_1_ (%pred)	87.78 ± 9.48	90.25 ± 7.81	0.091	-0.76–9.82
FEV_1_/FVC (%pred)	92.03 ± 13.60	89.30 ± 10.52	0.486	-10.59–5.13
PEF (%pred)	84.89 ± 11.58	81.55 ± 11.64	0.342	-10.37–3.69
FEF_25-75_ (%pred)	90.46 ± 22.68	87.90 ± 9.21	0.489	-9.98–4.86
Respiratory muscle strength				
MIP (cmH_2_O)	103.42 ± 23.49	100.90 ± 27.89	0.763	-19.32–14.28
MIP (%pred)	105.31 ± 22.97	95.10 ± 17.01	0.122	-23.28–2.86
MEP (cmH_2_O)	105.78 ± 29.94	112.70 ± 33.59	0.503	-13.80–27.64
MEP (%pred)	94.78 ± 19.82	90.65 ± 21.95	0.541	-17.68–9.42
Functional capacity				
6MWT distance (m)	605.84 ± 44.19	589.62 ± 34.57	0.087	-2.47–34.91
6MWT distance (%pred)	81.86 ± 5.09	78.85 ± 7.85	0.163	-1.27–7.29
ΔDyspnea (MBS)	0.89 ± 0.73	1.45 ± 1.09	0.073	-1.17–0.05
ΔFatigue (MBS)	0.71 ± 0.16	1.10 ± 0.24	0.179	-0.97–0.19
Minimum SpO_2_ (%)	95.31 ± 1.60	93.90 ± 1.74	0.071	-0.13–2.95
VEpeak (L/min)	32.56 ± 10.35	33.24 ± 4.59	0.811	-6.40–5.04
BR (%)	91.79 ± 2.76	89.21 ± 4.71	0.382	-3.33–8.49
RRpeak (1/min)	29.20 ± 12.38	31.03 ± 3.27	0.114	-1.30–3.64
ΔRR (1/min)	7.86 ± 6.44	5.74 ± 3.11	0.194	-1.13–5.37
ΔIC (ml)	-67.40 ± 43.10	-85.00 ± 49.97	0.314	-17.32–52.52

Data are presented as mean ± standard deviation

Abbreviations: *6MWT* 6-min walking test, *BR* breathing reserve, FEF_25–75_ forced mid-expiratory flow between 25% and 75%, FEV_1_ forced expiratory volume in 1 s, *FVC* forced vital capacity, *IC* inspiratory capacity, *MBS* modified Borg scale, *MEP* maximal expiratory pressure, *MIP* maximal inspiratory pressure, *PEF* peak expiratory flow, *RR* respiratory rate, T(dSpO2 ≥ %4): time spent with a drop of ≥ %4 in SpO2, *VE* minute ventilation

Table 3 summarizes the intragroup and intergroup comparisons of pulmonary function, respiratory muscle strength, and functional capacity between the EG and CG. No significant improvements were observed in FVC%, FEV_1_%, FEV_1_/FVC, PEF%, or FEF_25−75_% in either group (*p* > 0.05). Both groups showed significant improvements in MIP, MIP%, MEP, and MEP% (*p* < 0.001); however, intergroup differences were not statistically significant (*p* > 0.05). Regarding functional capacity, the 6MWT distance increased significantly in both groups (*p* < 0.001), with a significant group-by-time interaction favoring the EG (*p* = 0.024, ηp² = 0.127). Similarly, the percentage of predicted 6MWT distance improved in both groups (*p* < 0.05), though the intergroup difference was not significant (*p* = 0.096). The EG exhibited a significant increase in fatigue perception (*p* = 0.002), while dyspnea did not change significantly (*p* = 0.172). In contrast, the CG demonstrated a slight but significant reduction in fatigue (*p* = 0.049). The VE_peak_ significantly increased in the EG (*p* = 0.013) but not in the CG (*p* = 0.219), resulting in a significant group-by-time interaction (*p* = 0.048, ηp²=0.099). No significant changes were observed in the BR, RR_peak_, or ΔRR in either group (*p* > 0.05). A significant group-by-time interaction was found for ΔIC (*p* < 0.001, ηp²=0.933), indicating a greater improvement in inspiratory capacity and ventilatory response during exercise in the EG compared with the CG.

**Table 3:** Comparison of intragroup and intergroup differences in pulmonary function, respiratory muscle strength and functional capacity between the experimental and the control groups

			Experimental Group (*n* = 20)				Control Group (*n* = 20)		Intergroup differences**
	Baseline	Post-training	In-group Change (Δ)	Intragroup differences*	Baseline	Post-training	In-group Change (Δ)	Intragroup differences*	Time x Group
Pulmonary function									
FVC (% pred)	91.47 ± 9.78	91.91 ± 10.81	0.44 ± 2.56	*p* = 0.948 ηp^2^ = 0.002	96.00 ± 8.47	96.00 ± 8.83	0.00 ± 2.59	*p* = 1.000 ηp^2^ = 0.000	*p* = 0.620 ηp^2^ = 0.007
FEV_1_ (% pred)	87.78 ± 9.48	91.42 ± 10.76	3.64 ± 2.61	*p* = 0.052 ηp^2^ = 0.184	90.25 ± 7.81	91.75 ± 8.92	1.50 ± 2.45	*p* = 0.383 ηp^2^ = 0.040	*p* = 0.077 ηp^2^ = 0.071
FEV_1_/FVC (% pred)	92.03 ± 13.60	94.29 ± 14.41	2.26 ± 2.86	*p* = 0.265 ηp^2^ = 0.065	89.30 ± 10.52	89.50 ± 10.83	0.20 ± 2.21	*p* = 0.727 ηp^2^ = 0.007	*p* = 0.069 ηp^2^ = 0.083
PEF (% pred)	84.89 ± 11.58	88.49 ± 11.40	3.60 ± 2.48	*p* < 0.001 ηp^2^ = 0.584	81.55 ± 11.64	86.90 ± 11.51	5.35 ± 2.18	*p* < 0.001 ηp^2^ = 0.701	*p* = 0.113 ηp^2^ = 0.065
FEF_25-75_ (% pred)	90.46 ± 22.68	93.05 ± 21.97	2.59 ± 3.18	*p* = 0.074 ηp^2^ = 0.158	87.90 ± 9.21	89.20 ± 7.99	1.30 ± 2.15	*p* = 0.144 ηp^2^ = 0.109	*p* = 0.313 ηp^2^ = 0.027
Respiratory muscle strength									
MIP (cm H_2_O)	103.42 ± 23.49	111.68 ± 26.50	8.26 ± 25.06	*p* < 0.001 ηp^2^ = 0.952	100.90 ± 27.89	109.30 ± 29.94	8.40 ± 28.94	*p* < 0.001 ηp^2^ = 0.934	*p* = 0.984 ηp^2^ = 0.001
MIP (% pred)	105.31 ± 22.97	116.31 ± 25.45	11.00 ± 24.25	*p* = 0.002 ηp^2^ = 0.403	95.10 ± 17.01	99.15 ± 17.71	4.05 ± 17.36	*p* < 0.001 ηp^2^ = 0.971	*p* = 0.422 ηp^2^ = 0.017
MEP (cm H_2_O)	105.78 ± 29.94	123.21 ± 36.69	17.43 ± 33.59	*p* < 0.001 ηp^2^ = 0.928	112.70 ± 33.59	128.45 ± 33.76	15.75 ± 33.68	*p* < 0.001 ηp^2^ = 0.933	*p* = 0.911 ηp^2^ = 0.006
MEP (% pred)	94.78 ± 19.82	106.78 ± 27.91	12.00 ± 24.41	*p* < 0.001 ηp^2^ = 0.951	90.65 ± 21.95	97.45 ± 21.56	6.80 ± 21.75	*p* < 0.001 ηp^2^ = 0.952	*p* = 0.563 ηp^2^ = 0.009
Functional capacity									
6MWT distance (m)	605.84 ± 44.19	649.26 ± 33.65	43.42 ± 39.36	*p* < 0.001 ηp^2^ = 0.706	589.62 ± 34.57	599.27 ± 36.99	9.65 ± 35.80	*p* < 0.001 ηp^2^ = 0.690	*p* = 0.024 ηp^2^ = 0.127
6MWT distance (pred%)	81.86 ± 5.09	86.89 ± 8.90	5.10 ± 2.41	*p* < 0.001 ηp^2^ = 0.858	78.85 ± 7.85	79.32 ± 3.24	1.61 ± 5.24	*p* = 0.047 ηp^2^ = 0.195	*p* = 0.096 ηp^2^ = 0.071
ΔDyspnea (MBS)	0.89 ± 0.73	1.15 ± 0.95	0.31 ± 0.74	*p* = 0.172 ηp^2^ = 0.096	1.45 ± 1.09	1.25 ± 1.05	-0.20 ± 1.25	*p* = 0.483 ηp^2^ = 0.026	*p* = 0.283 ηp^2^ = 0.030
ΔFatigue (MBS)	0.78 ± 0.71	1.47 ± 1.11	0.68 ± 0.82	*p* = 0.002 ηp^2^ = 0.403	1.10 ± 0.24	0.74 ± 0.16	-0.35 ± 0.74	*p* = 0.049 ηp^2^ = 0.189	*p* = 0.062 ηp^2^ = 0.089
Minimum SpO2 (%)	95.31 ± 1.60	95.68 ± 1.29	0.36 ± 1.73	*p* = 0.368 ηp^2^ = 0.043	93.90 ± 1.74	95.25 ± 1.58	1.35 ± 2.27	*p* = 0.016 ηp^2^ = 0.269	*p* = 0.285 ηp^2^ = 0.030
VE_peak_ (L/min)	32.56 ± 10.35	36.35 ± 15.21	3.79 ± 12.90	*p* = 0.013 ηp^2^ = 0.283	33.24 ± 4.59	34.00 ± 4.65	0.76 ± 4.62	*p* = 0.219 ηp^2^ = 0.078	*p* = 0.048 ηp^2^ = 0.099
BR (%)	91.79 ± 2.76	91.48 ± 3.89	−0.31 ± 3.39	*p* = 0.742 ηp^2^ = 0.006	89.21 ± 4.71	89.78 ± 4.64	0.57 ± 4.67	*p* = 0.175 ηp^2^ = 0.095	*p* = 0.592 ηp^2^ = 0.008
RR_peak_ (1/min)	29.20 ± 12.38	29.91 ± 3.32	0.88 ± 2.89	*p* = 0.917 ηp^2^ = 0.001	31.03 ± 3.27	34.21 ± 16.26	3.01 ± 5.44	*p* = 0.786 ηp^2^ = 0.004	*p* = 0.447 ηp^2^ = 0.015
ΔRR (1/min)	7.86 ± 6.44	5.67 ± 2.97	−0.06 ± 2.16	*p* = 0.112 ηp^2^ = 0.118	5.74 ± 3.11	9.84 ± 7.20	1.97 ± 5.55	*p* = 0.895 ηp^2^ = 0.001	*p* = 0.271 ηp^2^ = 0.032
ΔIC (ml)	-67.40 ± 43.10	-5.50 ± 30.86	30.00 ± 37.43	*p* = 0.724 ηp^2^ = 0.007	-85.00 ± 49.97	24.79 ± 24.84	180.05 ± 42.43	*p* = 0.080 ηp^2^ = 0.153	*p* < 0.001 ηp^2^ = 0.933

Data are presented as mean ± standard deviation

Abbreviations: 6MWT: 6-min walking test, BR: breathing reserve, FEF25–75: forced mid-expiratory flow between 25% and 75%, FEV1: forced expiratory volume in 1 s, FVC: forced vital capacity, IC: inspiratory capacity, MBS: modified Borg scale, MEP: maximal expiratory pressure, MIP, maximal inspiratory pressure, PEF: peak expiratory flow, RR: respiratory rate, VE: minute ventilation

*Repeated-measures ANOVA

**Mixed ANOVA

## Discussion

The key finding of this study was that both the novel individualized respiratory exercise device and the conventional RMT devices effectively improved respiratory muscle strength and functional capacity in healthy adults. However, the novel individualized device provided greater improvements in 6MWT distance, VEpeak, and ΔIC, indicating enhanced ventilatory efficiency and a more favourable ventilatory response during exercise. Both interventions significantly increased MIP and MEP values; however, pulmonary function parameters, including FVC, FEV_1_, and FEF_25−75_, remained unchanged. This finding aligns with previous studies indicating that respiratory muscle training predominantly enhances muscle performance rather than static lung volumes. The observed changes in ΔIC may reflect improved inspiratory capacity preservation during exertion rather than true dynamic hyperinflation, which is typically associated with obstructive lung diseases. These results collectively suggest that the novel device’s individualized resistance adjustment and real-time feedback features may optimize training effectiveness and ventilatory mechanics even in individuals without pulmonary impairment.

Although both interventions resulted in improvements in respiratory muscle strength, no significant changes were observed in FVC, FEV₁, or FEF₂₅–₇₅ values in either group, while PEF showed a significant increase within both groups, with no intergroup difference. Similar findings have been reported in previous studies examining respiratory muscle training in healthy adults, where improvements were primarily seen in flow-dependent parameters such as PEF rather than static volumes like FVC or FEV_1_ [3, 17]. This selective enhancement in expiratory flow may be attributed to the greater recruitment and strengthening of expiratory muscles, particularly the abdominal and internal intercostal muscles, which play a key role in generating higher expiratory pressures during forced breathing maneuvers [23]. The absence of significant changes in other spirometric indices may be related to the normal baseline pulmonary function of healthy participants, leaving limited potential for measurable improvement [2]. Furthermore, previous studies have suggested that short-term respiratory muscle training protocols, typically lasting less than 12 weeks, primarily induce neuromuscular adaptations—such as improved motor unit synchronization and increased respiratory muscle efficiency—rather than structural changes that could significantly alter lung volumes [24, 25]. Taken together, these results suggest that the observed improvement in PEF likely reflects enhanced expiratory muscle performance rather than true alterations in lung mechanics, and that both IMT and EMT may have contributed equally to this outcome through similar resistance-based loading mechanisms.

Both training modalities in the present study led to significant improvements in inspiratory and expiratory muscle strength, as evidenced by increased MIP and MEP values in both groups. These results are consistent with previous research demonstrating that respiratory muscle training enhances respiratory muscle performance in healthy adults and athletes, regardless of the specific training device employed [17, 23]. The magnitude of improvement observed in this study aligns with the findings of Enright and Unnithan, who reported significant increases in respiratory muscle strength following an 8-week inspiratory muscle training program at 40% of maximal inspiratory pressure in healthy individuals [17]. Similarly, Sales et al. [23] found that both athletes and non-athletes exhibited greater respiratory muscle endurance and strength after training, suggesting that RMT-induced adaptations occur independently of baseline fitness levels. The comparable strength gains between groups indicate that both the novel individualized device and the conventional devices provided sufficient mechanical load to stimulate muscle adaptation. However, the individualized device’s integrated inspiratory and expiratory loading, combined with real-time visual feedback, may have facilitated better breathing pattern control and enhanced user engagement. Visual feedback has been shown to promote greater adherence and motor learning by providing immediate information about performance quality, thereby improving neuromuscular coordination and training efficiency [6]. Additionally, the individualized adjustment of resistance likely contributed to maintaining optimal training intensity throughout the intervention, preventing under- or overloading and ensuring consistent stimulus for adaptation [8]. Collectively, these mechanisms may explain the observed gains in respiratory muscle strength and the superior ventilatory response achieved by the EG.

In the present study, both groups demonstrated significant increases in 6MWT distance following the 8-week training, with greater gains in the EG. The 6MWT is a well-validated measure of submaximal functional exercise capacity that reflects the integrated responses of respiratory, cardiovascular, and musculoskeletal systems [26]. Improvements in 6MWT distance have been frequently observed following RMT in both healthy and clinical populations [2, 27]. These improvements are generally attributed to enhanced respiratory muscle strength and endurance, leading to a reduction in the work of breathing and improved oxygen delivery to peripheral muscles [28]. Recent studies in healthy adults have reported significant gains in functional exercise capacity after RMT protocols ranging from 6 to 12 weeks, suggesting that respiratory muscle loading can enhance submaximal performance even in the absence of pulmonary impairment [17, 24]. Similarly, IMT has been shown to improve walking distance and exercise tolerance in overweight and obese adults [29]. These results are consistent with our findings and suggest that the individualized device, by providing simultaneous inspiratory and expiratory resistance and real-time feedback, may enhance training adherence, breathing coordination, and overall performance efficiency. The greater improvement in 6MWT distance in the EG highlights the potential role of individualized RMT in augmenting general physical capacity among healthy individuals. Furthermore, it should be noted that a learning effect associated with repeated 6MWT assessments may have contributed to the observed improvements in walking distance. In addition, both groups were advised to engage in regular walking activity during the intervention period, which may have further influenced functional outcomes. However, as these factors were applied equally to both groups, they are unlikely to explain the greater improvement observed in the EG.

Beyond walking distance, ventilatory parameters such as peak minute ventilation (VE_peak_) and the change in inspiratory capacity (ΔIC) provide additional insight into the ventilatory response to exercise. In this study, VE_peak_ increased significantly only in the EG, while ΔIC exhibited a significant group-by-time interaction, indicating improved ventilatory efficiency and inspiratory muscle performance. These findings are consistent with evidence that RMT can improve ventilatory control and reduce respiratory muscle fatigue during exercise in healthy populations [8, 30]. A reduction in ΔIC during exertion is known to reflect dynamic hyperinflation, a phenomenon associated with increased end-expiratory lung volume and diminished inspiratory reserve capacity [31]. Although dynamic hyperinflation has been primarily studied in patients with chronic obstructive pulmonary disease, similar mechanical constraints can transiently occur in healthy adults during high ventilatory demand, leading to a higher work of breathing and earlier onset of dyspnea [32, 33]. RMT has been shown to mitigate these effects by improving respiratory muscle strength and breathing pattern regulation, allowing more efficient ventilation at a given workload [1, 34]. The individualized respiratory device used in this study likely optimized ventilatory mechanics through its simultaneous inspiratory–expiratory resistance design and visual feedback, resulting in superior improvements in VE_peak_ and ΔIC and a reduced tendency toward dynamic hyperinflation. These adaptations suggest enhanced ventilatory economy and mechanical efficiency following individualized RMT.

The increase in perceived fatigue observed in the EG may be attributed to the higher ventilatory demand associated with dual-resistance training and the use of real-time visual feedback, which may have encouraged greater effort during exercise [2, 8]. In contrast, the reduction in fatigue in the CG may reflect adaptation to a more familiar and less demanding training modality. It is also important to consider that fatigue perception is subjective and may be influenced by increased awareness of breathing effort, particularly in the presence of feedback [6]. Therefore, the observed increase in fatigue in the EG may indicate a higher level of exertion rather than reduced efficiency.

The findings of this study provide meaningful insights into the optimization of RMT in healthy adults. The significant improvements in respiratory muscle strength, ventilatory efficiency, and functional capacity observed in both groups support the efficacy of RMT even in non-clinical populations. Importantly, the superior outcomes achieved with the novel individualized respiratory exercise device highlight the potential benefits of integrating both inspiratory and expiratory muscle training within a single system, coupled with real-time visual feedback and individualized load adjustment. Such a design not only enhances user engagement and training adherence but also ensures a progressive and optimal mechanical load, which is essential for long-term muscle adaptation. These features may make the device particularly useful in preventive, sports, and early rehabilitation settings where improving ventilatory control and endurance is desired.

However, several limitations should be acknowledged. First, the study sample consisted solely of healthy adults, which limits the generalizability of the results to clinical populations such as individuals with pulmonary or cardiovascular conditions. Additionally, the sample size calculation was based on a correlation coefficient rather than a between-group effect size, which may limit the precision of the estimation for a randomized controlled design. Second, the intervention period was limited to 8-weeks; while this duration was sufficient to elicit functional and ventilatory adaptations, structural respiratory muscle changes may require a longer training period of at least 12-weeks, as suggested by previous research. Third, while adherence was monitored through exercise diaries, direct supervision during home sessions was not feasible, which might have introduced minor variations in training quality and effort. Fourth, the study did not include advanced physiological measurements such as electromyography, oxygen kinetics, or dyspnea perception analysis that could provide deeper insights into the mechanisms underlying the observed adaptations. Fifth, the recommendation to engage in regular walking activity may be considered a limitation. However, this advice was provided equally to both groups as part of general physical activity recommendations aimed at promoting healthy behavior during the intervention period. A further limitation of the study is that participants’ views on the real-time feedback feature were not assessed with a structured or validated method. Although some participants verbally stated that the visual feedback was motivating during exercise, these comments were informal and should be interpreted with caution. Future studies should address these limitations by incorporating longer intervention periods, diverse populations, and objective physiological outcome measures to further validate the device’s clinical utility.

## Conclusion

In conclusion, this study demonstrated that both conventional and novel individualized respiratory exercise devices effectively improved respiratory muscle strength and functional capacity in healthy adults, while the novel device produced superior enhancements in ventilatory performance and inspiratory capacity. These findings indicate that individualized, dual-resistance respiratory training with visual feedback and individualized load adjustment may offer a more efficient and engaging approach to optimizing ventilatory mechanics and overall physical performance. The novel device thus represents a promising, clinically applicable innovation for use in both health promotion and rehabilitation programs.

## Data Availability

The data that support the findings of this study are available from the corresponding author upon reasonable request.
